# Diagnosing popliteofibular ligament injuries in anterior cruciate ligament‐injured knees: A prospective magnetic resonance imaging study investigating the inter‐ and intraobserver reliability of identification of the popliteofibular ligament

**DOI:** 10.1002/jeo2.12112

**Published:** 2024-07-24

**Authors:** Steven Heylen, Thomas Braeckevelt, Peter Verdonk, Matthias Krause, Jozef Michielsen

**Affiliations:** ^1^ Department of Trauma and Orthopaedics Heilig Hart Ziekenhuis Lier Lier Belgium; ^2^ Orthopaedic Research and Education Foundation OrthoClinic Lier Lier Belgium; ^3^ PhD Department, Faculty of Medicine and Health Sciences University of Antwerp Antwerp Belgium; ^4^ OrthoCA Orthopaedic Center Antwerp Belgium; ^5^ Department of Orthopaedic Surgery Antwerp University Hospital Edegem Belgium; ^6^ Department of Trauma Surgery and Orthopaedics UKE Hamburg Germany

**Keywords:** knee instability, popliteofibular ligament, posterolateral corner

## Abstract

**Purpose:**

The aim of our study was to investigate the intra‐ and interobserver reliability for the identification of the popliteofibular ligament (PFL) in magnetic resonance imaging (MRI) scans in patients with an anterior cruciate ligament (ACL) injury and ascertain the prevalence of PFL tears in ACL‐injured knees without clinically high‐grade posterolateral corner injury.

**Methods:**

MRI readings were performed retrospectively by two surgeons on 84 patients who underwent ACL reconstruction in our department. The presence of the PFL on both sagittal and coronal images as well as the presence of PFL tears was noted. Readings were repeated 6 weeks later for one observer. The *κ* value was calculated to determine the intra‐ and interobserver reliability for identification of the PFL and the prevalence of PFL tears was ascertained.

**Results:**

The PFL was visualized in 90.5%−91.7% of MRI scans. The intra‐ and interobserver reliability of visualizing the PFL on MRI had an *κ* value of 0.63 and 0.66 (substantially reliable), respectively. The intraobserver reliability for identification of PFL tears had an *κ* value of 0.26 (fair reliability). We found a 4.8% prevalence of PFL tears in ACL‐injured knees.

**Conclusions:**

There is substantially reliable intra‐ and interobserver reliability for the identification of the PFL on MRI scans but only fair reliability for the identification of PFL tears. A 4.8% prevalence of PFL tears in ACL‐injured knees without clinically confirmed high‐grade posterolateral corner injury can be observed in our series.

**Level of Evidence:**

Level IV.

**Abbreviations**
ACL: anterior cruciate ligament
CI: confidence interval
LCL: lateral collateral ligament
MCL: medial collateral ligament
MRI: magnetic resonance imaging
PFL: popliteofibular ligament
PLC: posterolateral corner
PT: popliteus tendon

## INTRODUCTION

**Figure 1:**
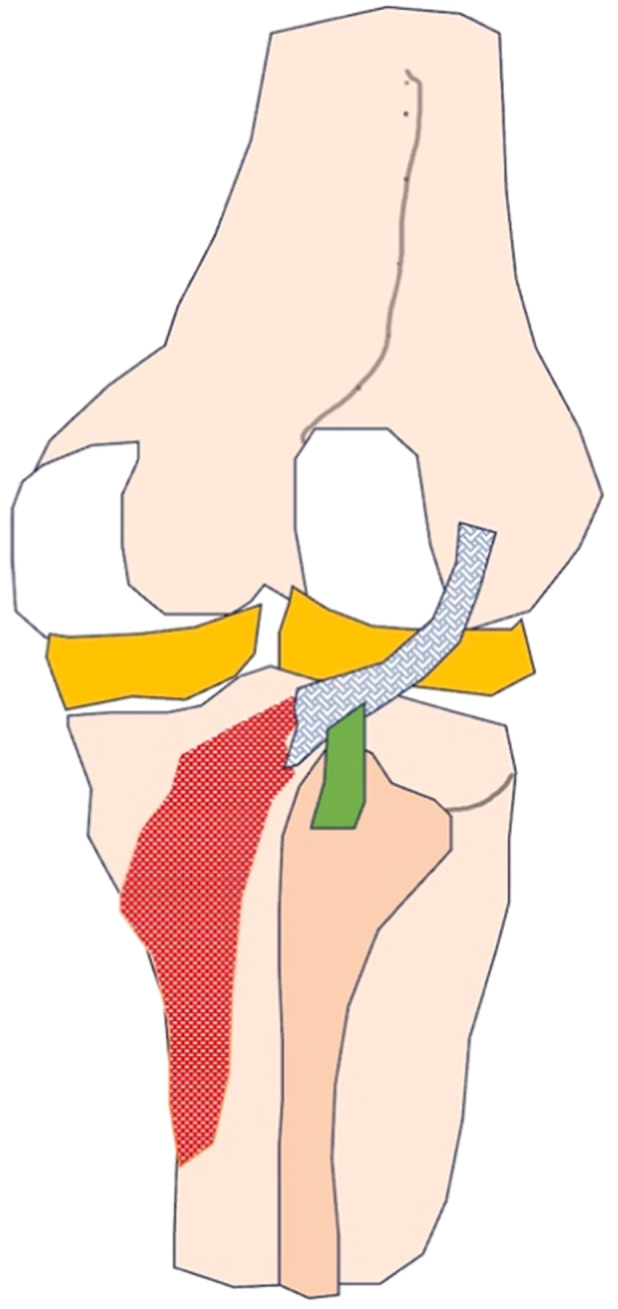
The popliteofibular ligament (green) and popliteus tendon (grey).

Posterolateral corner injuries (PLC) occur in anterior cruciate ligament (ACL) tears in 5%−19.7% of cases [2, 5, 16, 20, 21, 22, 26, 30, 34, 36]. Type A PLC injuries involve only the popliteofibular ligament (PFL) and/or popliteus tendon (PT) (Figure 1). Type A PLC injuries are underreported in the literature. The diagnosis of type A injuries is difficult because only an increase in external rotation can be observed clinically in these cases. In the acute setting, pain can be a limiting factor in the clinical diagnosis of these injuries, preventing accurate assessment of the increase in external rotation of the tibia [4, 9, 11, 17, 20, 25, 29]. Many authors have therefore suggested a thorough magnetic resonance imaging (MRI) investigation of all PLC structures in injured knees [8, 26, 33, 34, 35]. Routine evaluation of the PFL on MRI scans, however, is not common with papers mentioning a reporting frequency of less than 50% [8, 11, 32]. We found that only one study had published reporting on the interobserver reliability of the PFL on MRI scans in ACL‐injured knees [11]. Diagnosing PFL tears on MRI scans could be important to alert the clinician for a possible type A PLC injury. Currently, it is unclear in the literature whether the PFL should be assessed on every MRI scan of an injured knee, and many studies investigated this subject in high‐grade (type B and C) PLC injuries instead of type A PLC injuries. The prevalence of PFL tears and thus possible type A PLC injuries in patients with an ACL tear is also underreported in the literature. We found only four studies reporting on PFL tears in combination with ACL tears, and the prevalence published in the literature has a wide range [17, 21, 23, 35].

The first aim of our study is to determine the inter‐ and intraobserver reliability for the identification of the PFL on coronal and sagittal MRI images in patients with an ACL injury. The second aim is to ascertain the prevalence of PFL injuries in ACL‐injured knees without clinically high‐grade PLC injury in our series. Our hypothesis is that the PFL can be identified on MRI scans in patients with an ACL injury with at least a moderate intra‐ and interobserver reliability and that the findings of a PFL tear on MRI scans in our series of ACL‐injured knees without clinically high‐grade PLC injury is rare (<5%).

## METHODS

The study was registered with the local ethical committee and approved. In a retrospective study, the MRI scans of 150 patients who underwent ACL reconstructive surgery in our department in 2021−2023 were evaluated. Exclusion criteria were prior knee surgery, prior knee fractures, clinically confirmed PLC injury in combination with an ACL reconstruction and MRI scans more than 4 weeks after the injury. The following patient characteristics were assessed: age at time of injury and gender.

MRI scans were performed in a Philips 3T MRI machine. Proton density‐weighted spectral presaturation with inversion recovery sequences in the sagittal, axial and coronal planes were obtained, and in the sagittal plane, an additional T1‐TSE sequence was obtained. The evaluation was performed using an Xero Viewer version 8.2.0.160 (Agfa Healthcare).

**Figure 2:**
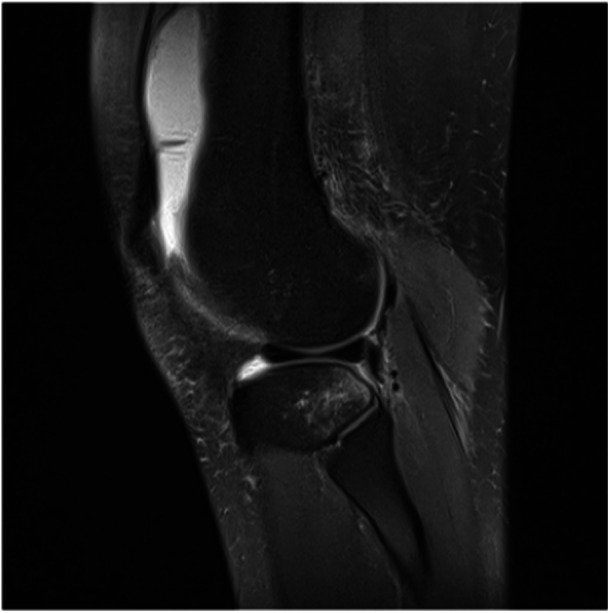
Magnetic resonance imaging scan with popliteofibular ligament tear.

The presence of the PFL was noted on coronal and sagittal images separately and pooled. Readings were performed by a sports medicine‐trained knee surgeon and an orthopaedic surgery resident. Both readers were blinded from the readings of the other and from any patient information that could influence the reading. We chose to perform our readings in surgically confirmed ACL‐injured knees and MRI scans less than 4 weeks after trauma to replicate MRI investigation in the clinical setting with intraarticular haematoma and oedema in the posterolateral tissues due to the ACL injury and pivot shift phenomena. PFL sprains and tears were graded according to their appearance on sagittal and coronal MRI images, as previously described in the literature [18]. The images were assessed for the presence of a PFL tear, which was defined as complete discontinuity of ligament fibres (Figure 2). Only in case of complete discontinuity of the ligament fibres, the PFL was noted to be torn. The PFL was noted to be torn if one or both of the readers marked the PFL as torn. Injuries to other structures of the PLC, such as the lateral collateral ligament and PT, were also noted. In cases of the irregular contour of the PFL or (peri‐)ligamentous oedema with continuity of the ligament, the ligament status was deemed intact, as mentioned in other studies [4]. The prevalence of complete PFL tears was calculated. Six weeks later, the readings of one observer were repeated by the same sports medicine‐trained knee surgeon to determine the intraobserver reliability of the identification of the PFL. These readings were performed without any information on the results of the last readings and blinded from any patient information that could influence the reading.

The *κ* value was calculated to determine the intraobserver reliability. *κ* values between 0 and 0.20 indicate poor reliability; values between 0.21 and 0.4 indicate fair reliability; values between 0.41 and 0.6 indicate moderate reliability; values between 0.61 and 0.80 indicate substantial reliability; and values between 0.81 and 1.00 indicate excellent reliability as previously mentioned in the literature [19]. The statistical analysis was performed using SPSS 21.0 (SPSS Inc.). The difference in visualization of the PFL on coronal and sagittal images was determined with a chi‐square test.

## RESULTS

**Figure 3:**
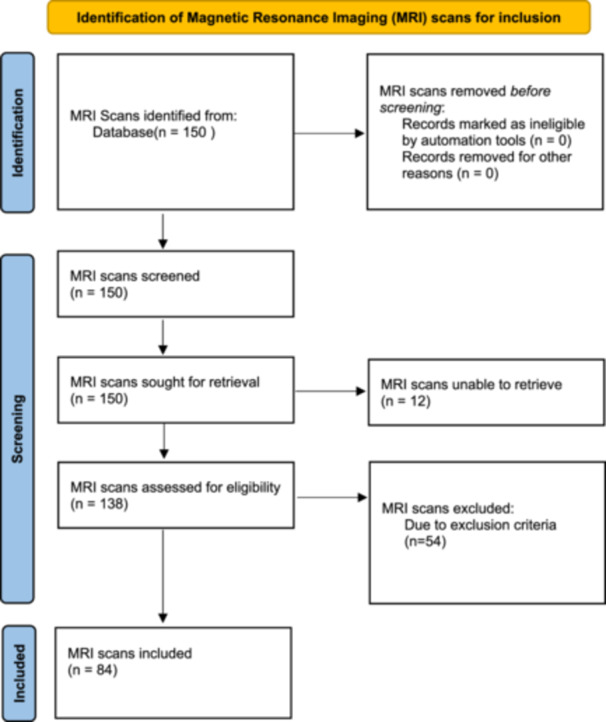
Flowchart for identification of MRI scans to be included in the study. MRI, magnetic resonance imaging.

A total of 150 patients who underwent ACL reconstruction in our department in 2021−2023 were evaluated in a retrospective study format. After the application of the exclusion criteria, 84 MRI scans in 84 patients were available for assessment. The main cause for exclusion was the timing of the MRI scan. The mean age of the patients was 27.5 years (range: 14−54 years). Fifty MRIs were of the right knee, and 34 MRIs were of the left knee. Sixty‐four patients were male, and 20 patients were female. The mean time from injury to the MRI scan was 16 days (range: 2−28 days). Table 1 summarizes the study characteristics. Table 2 summarizes the results of the study. Figure 3 depicts the flowchart for MRI inclusion. In 8 (9.5%) patients, the PFL could not be visualized on sagittal or coronal images for Observer 1 and in 7 (8.3%) patients for Observer 2. Observer 1 identified 4 (4.8%) patients in which the PFL was torn with complete loss of fibre continuity. In three of these four patients, Observer 2 marked the PFL as invisible but did not make a statement concerning a tear. In one of these patients, Observer 2 marked the PFL as intact and visible. In 2 (2.4%) patients, there was an obvious distal LCL injury with loss of continuity of the LCL. In these two patients, there was no PFL tear. We found bone marrow oedema in two patients in the fibular head. In one of those patients, the distal LCL was torn. The PFL was not torn in these two patients. The PFL could not be visualized on the coronal MRI image in 35 (41.6%) patients (Observer 1) and 39 (44.8%) patients (Observer 2) and could not be visualized on the sagittal MRI image in 20 (23%) (Observer 1) and 8 (9.5%) (Observer 2) patients. We found a statistically significant difference in the ability to visualize the PFL on the coronal versus sagittal images (*p* = 0.03). The intraobserver reliability for visualizing the PFL on MRI between the first and second review of the MRIs had an *κ* value of 0.63 (CI: 0.41−0.91), which is considered substantially reliable. The intraobserver reliability for identification of a PFL tear between the first and second review of the MRIs had an *κ* value of 0.26 (CI: 0.14−0.36), which is only fair reliability. The interobserver reliability of visualizing the PFL on coronal and/or sagittal MRI images was substantially reliable (*κ* = 0.66; CI: 0.31−0.89).

**Table 1:** Study information.

Number of MRI scans evaluated	84
Male/female patients	64 males/20 females
Mean age of patients	27.5 years (range: 14−54 years)
Mean time to MRI scan	16 days (range: 2−28 days)

Abbreviation: MRI, magnetic resonance imaging.

**Table 2:** Study results.

Percentage of PFL identification	Observer 1	Observer 2
Combined sagittal–coronal	90.5%	91.7%
Sagittal	77%	90%
Coronal	58.4%	55.2%
Interobserver reliability for visualization PFL	Kappa value = 0.66 (CI: 0.31−0.89)	
Intraobserver reliability for visualization PFL	Kappa value = 0.63 (CI: 0.41−0.91)	/
Intraobserver reliability PFL tears	Kappa value = 0.26 (CI: 0.14−0.36)	/
Percentage of PFL tears in ACL patients	4.8%	0%

Abbreviations: ACL, anterior cruciate ligament; PFL, popliteofibular ligament.

## DISCUSSION

The most important finding of this study was that Observer 1 and Observer 2 were able to identify the PFL in 90.5% and 91.7% of cases, respectively, with a statistically significant difference in the ability to visualize the PFL on the coronal versus sagittal images (*p* = 0.03). These reported percentages are in line with those reported by others [23, 27]. The MRI scans in our series were reviewed by a sports medicine‐trained knee surgeon and an orthopaedic surgery resident, but the percentages match those reported in the literature, where the images were reviewed by radiologists specializing in musculoskeletal imaging. It is clear from our results that the PFL should be reviewed on both sagittal and coronal images. We found substantial reliability for visualizing the PFL on MRI scans but only fair reliability for diagnosing PFL tears. This finding is in line with the same finding about the anterolateral ligament of the knee, which is another thin structure of the knee [13]. The overall prevalence of PFL tears in patients with a known ACL tear appears to be low. We found only a 4.8% prevalence of PFL tears in ACL‐injured knees without clinically confirmed high‐grade PLC injury in our series. It is unclear whether these PFL tears on MRI are in fact part of a type A PLC injury as we do not have clinical data to support this.

### Diagnosing type A PLC injuries

In type A PLC injuries, the PFL and/or PT are injured, and these patients only exhibit an increased external rotation on clinical examination. In the acute setting, pain can be a limiting factor in the clinical diagnosis of these injuries, preventing accurate assessment of the increase in external rotation of the tibia [4, 9, 11, 17, 20, 25, 29]. ACL injuries are attributed to an increase in external rotation of up to 7° further complicating the diagnosis of type A PLC injuries [12]. Many authors have therefore suggested a thorough MRI investigation of all PLC structures in ACL‐injured knees [8, 26, 33, 34, 35]. In a recent expert consensus study, Chahla et al. concluded that experts believe that MRI should always be performed in the assessment of suspected acute PLC injuries, but they do not specify which structures should be evaluated [6]. In the acute setting, MRI evaluation of certain structures is more difficult due to soft tissue oedema [3, 8, 15, 27]. The literature is inconsistent regarding the accuracy of MRI in evaluating smaller PLC structures, such as the PFL [7, 10, 14, 21, 29]. We found that reliable identification of the PFL on MRI scans seems to be possible, but reliable identification of PFL tears appears more difficult. Rakhra et al. found a high accuracy of MRI for detecting tears of the PLC components (lateral collateral ligament, biceps femoris tendon, PT and posterolateral ligamentocapsular complex), ranging from 82% to 95% in multiligament‐injured knees but did not evaluate the PFL as a separate structure [29]. In a large study evaluating 178 MRIs in patients with multiligamentous knee injuries, the reporting percentage of the PFL was only 47.8% [32]. The diagnostic accuracy, however, was 80.7%, equal to that of the LCL, but the LCL had a reporting percentage of 94.9%. The positive predictive value of the PFL in the study by Sanchez‐Munoz et al. was 80.8%, almost equal to that of the medial collateral ligament (MCL), but the MCL had a reporting percentage of 99.4% [32]. McKean et al. found a sensitivity for visualization of the PFL of 90% and they mention that routine review of the PLC and PFL should be an essential part of acute knee imaging [23]. Other authors mention that the increased sensitivity of current imaging modalities can potentially cause overreporting of injuries that are not clinically significant, and in many MRI studies, the clinical significance of these injuries is not reported [1, 24, 31]. This is also the case in our study. In contrast to the conclusions of McKean et al. [23], Filli et al. reported only a 53.1%−63% sensitivity for identification of the PFL on MRI studies in patients with an ACL injury [11]. The interobserver agreement for the evaluation of PFL integrity in their study was only fair. They concluded that assessment of the PFL on MRI does not add to the diagnostic accuracy of PLC injuries. Other authors emphasize the same conclusion, but many of these studies investigated type B or C PLC injuries. Obviously, in higher‐grade PLC injuries with high‐grade ruptures of the LCL and PT, MRI investigation of smaller PLC structures becomes redundant. In lower‐grade PLC injuries without LCL injuries (such as in type A PLC injuries), MRI investigation of smaller PLC structures could be more important, and the results of our study show that with substantial intra‐ and interrater agreement, the PFL can be evaluated on MRI scans in cases of traumatic knee injury. Pękala et al. underline the strong need to improve MRI assessments of the PFL, which is a crucial step in the diagnosis of PLC injuries and in developing treatment plans [27]. To increase the likelihood of visualizing the PFL on MRI imaging, other sequences and MRI techniques have been investigated, but none are routinely used in most departments [28, 37].

### Percentage of PFL tears in ACL‐injured knees

Few studies actually report on PFL tears in MRIs in ACL‐injured or multiligamentous injured knees. Temponi et al. report 4.3% PFL tears in their patients with ACL injury, which is in line with the results of our study [35]. Kinsella et al. found 10% of PFL injuries in ACL injuries in a paediatric population, and this was the most common combination for ACL–PLC injury in their series [17]. McKean et al. found 20% of torn PFLs in acutely traumatic injured knees, and they found 28.4% of PFL injuries in ACL‐injured patients [23]. Laprade et al. found an incidence of 1.2% isolated PFL tears in acute knees with hemarthrosis [21]. They only reported on high‐grade PFL tears. They found only two patients with an ACL injury combined with an isolated PFL injury. This is only 1.5% of all ACL‐injured patients. However, they did find a 7.4% combination of ACL–PLC injuries. The percentage of PFL tears in ACL tears without obvious high‐grade clinical PLC injuries seems to be underreported in the literature with PFL tears occurring in the setting of an ACL tear reported in a wide range of 1.5%−28.4% of cases [21, 23]. We found PFL tears in ACL‐injured patients in only 4.8% of our patients.

We acknowledge certain limitations to this study. Due to the rather rare combination of PFL tears in ACL‐injured patients, our total of 84 MRI scans could potentially be low, and this could have an influence on the percentage of PFL tears because of the limited statistical power. A further limitation is the absence of clinical examination in these patients. In particular, it would be very useful to ascertain whether the patients with a PFL tear on an MRI scan indeed exhibited increased external rotation during clinical examination. The lack of data on the clinical examination implies that it is impossible to correlate these PFL tears with clinical type A PLC instability. The retrospective nature of the study could potentially cause some inherent bias due to the aim of the study. The timing of the MRI scan (less than 4 weeks from injury) could also lead to a false‐positive result regarding the reading of a PFL tear. However, we aimed to reduce this limitation by only reporting grade III PFL signals as a tear.

## CONCLUSIONS

We found a substantially reliable intra‐ and interobserver reliability for the identification of the PFL in MRI scans but only fair reliability for the identification of PFL tears. We found a 4.8% prevalence of PFL tears in ACL‐injured knees without clinically confirmed high‐grade PLC injury in our series.

## AUTHOR CONTRIBUTIONS

*Manuscript preparation*: Steven Heylen. Otherwise, equal among authors.

## CONFLICT OF INTEREST STATEMENT

The authors declare no conflict of interest.

## ETHICS STATEMENT

Ethical approval was obtained from the local ethical committee.

## Data Availability

The data that support the findings of this study are available from the corresponding author, upon reasonable request.
